# 127 Diagnostic Stewardship: Reducing Urine Cultures by requiring an indication

**DOI:** 10.1017/ash.2026.10539

**Published:** 2026-06-23

**Authors:** Anna Pałka, Mateusz Gajda, Estera Jachowicz-Matczak, Jadwiga Wókowska-Mach

**Affiliations:** 1 Chair of Microbiology Medical Faculty Jagiellonian University Medical College, Kraków Poland; 2 Jagiellonian Univeristy / Univeristy Hospital in Cracow; 3 Collegium Medicum Jagiellonian University; 4 Jagiellonian University Medical School

## Abstract

**Background:** Clostridioides difficile infection (CDI) remains a major healthcare challenge, particularly in resource-limited settings. **Methods:** This retrospective, single-centre study analyzed CDI epidemiology and treatment outcomes among 528,887 hospitalized patients at the University Hospital in Kraków Poland, between 2016 and 2022. **Results:** A total of 2,341 CDI cases were confirmed, with an overall incidence of 4.32 per 1,000 admissions. The highest rates were observed in geriatric and infectious diseases units. During the COVID-19 pandemic, healthcare-associated CDI cases surged, accounting for up to 89.2% of infections in 2020 with incidence rate 3.8 per 1000 admissions, compare with 2,5 per 1000 admissions in 2016. Vancomycin-based therapy was associated with significantly lower mortality (OR 0.73, 95% CI 0.56-0.95) compared to metronidazole, while combination therapy (vancomycin + metronidazole) showed the highest recurrence rate (17%). Fidaxomicin use was minimal (0.4%) due to limited availability. Recurrent CDI occurred in 14.2% of cases, with a relapse-free survival advantage observed in vancomycin-treated patients. The overall in-hospital case fatality rate associated with CDI was 22.5%. **Conclusions:** Despite stable overall CDI incidence, the study highlights the impact of increased antibiotic consumption during the pandemic on HA-CDI dynamics. Findings underscore the need for improved antimicrobial stewardship, broader access to advanced therapies such as fidaxomicin and bezlotoxumab, and enhanced diagnostic protocols. In settings with restricted therapeutic options, vancomycin remains a valuable treatment, particularly for reducing mortality.

